# Psychological risk and protective factors for disability in chronic low back pain – a longitudinal analysis in primary care

**DOI:** 10.1186/s12891-017-1482-8

**Published:** 2017-03-20

**Authors:** Nikita Roman A. Jegan, Markus Brugger, Annika Viniol, Konstantin Strauch, Jürgen Barth, Erika Baum, Corinna Leonhardt, Annette Becker

**Affiliations:** 1 Department of General Practice and Family Medicine, Philipps-University Marburg, Karl-von-Frisch-Str. 4, 35032 Marburg, Germany; 2 Institute of Medical Informatics, Biometry and Epidemiology, Chair of Genetic Epidemiology, Ludwig-Maximilians-Universität, Munich, Germany; 3 Institute of Genetic Epidemiology, Helmholtz Zentrum München – German Research Center for Environmental Health, Neuherberg, Germany; 4 Institute for Complementary and Integrative Medicine, University Hospital Zurich und University Zurich, Zurich, Switzerland

**Keywords:** Chronic low back pain, Longitudinal study, Primary care, Resilience, Self-efficacy

## Abstract

**Background:**

Utilizing psychological resources when dealing with chronic low back pain might aid the prevention of disability. The observational study at hand examined the longitudinal impact of resilience and coping resources on disability in addition to established risk factors.

**Methods:**

Four hundred eighty four patients with chronic low back pain (>3 months) were recruited in primary care practices and followed up for one year. Resilience, coping, depression, somatization, pain and demographic variables were measured at baseline. At follow-up (participation rate 89%), data on disability was collected. We first calculated bivariate correlations of all the predictors with each other and with follow-up disability. We then used a multiple regression to evaluate the impact of all the predictors on disability together.

**Results:**

More than half of the followed up sample showed a high degree of disability at baseline (53.7%) and had suffered for more than 10 years from pain (50.4%). Besides gender all of the predictors were bivariately associated with follow-up disability. However in the main analysis (multiple regression), disability at follow up was only predicted by baseline disability, age and somatization. There was no relationship between resilience and disability, nor between coping resources and disability.

**Conclusions:**

Although it is known that there are cross-sectional relationships between resilience/coping resources and disability we were not able to replicate it in the multiple regression. This can have several reasons: a) the majority of patients in our sample were much more disabled and suffered for a longer time than in other studies. Therefore our results might be limited to this specific population and resilience and coping resources might still have a protective influence in acute or subacute populations. b) We used a rather broad operationalization of resilience. There is emerging evidence that focusing on more concrete sub facets like (pain) self-efficacy and acceptance might be more beneficial.

**Trial registration:**

German Clinical Trial Register, DRKS00003123 (June 28th 2011).

**Electronic supplementary material:**

The online version of this article (doi:10.1186/s12891-017-1482-8) contains supplementary material, which is available to authorized users.

## Background

Disability is one of the most important complications of chronic low back pain (CLBP). In a German study based on the general population, about 16% of individuals with pain reported high levels of disability [1]. This is even higher in patient based studies where up to 60% [2] of those suffering from CLBP report high disability. Psychological risk factors for chronification and the development of disability are well researched and implemented in guidelines for the management of chronic pain [3–7]. For example the European guidelines for the management of CLBP mention distress, depression, pain severity, functional impact (disability), cognitions, extreme symptom report (somatization) and prior pain episodes besides work related factors [7]. The German National Disease Management Guideline for Low back pain also reports depression, distress, a tendency towards somatization and pain-related cognitions but also adds overactive suppressive pain behavior [5].

Less is known about protective factors possibly leading to favourable courses of back pain. This is especially important since the mere absence of risk factors does not predict successful adaptation to chronic pain [8]. Additionally interventions targeted towards specific risk factors are only rarely better than usual care [9]. Therefore focusing on constructs from positive psychology such as resilience or coping resources might identify protective factors which can be implemented in the management of chronic pain and promote successful adaptation [10].

Resilience is an important construct in health research and positive psychology. It refers to the successful adaptation to adverse experiences which can be singular such as a trauma or continuous such as chronic pain. The construct itself however is heterogeneous and there is disagreement considering its exact definition [11, 12]. Some understand it as qualities inherent in the individual (i.e. a stable personality trait), whereas others focus on the reactions to an adverse experience (i.e. resilient processing) or on the outcome of adaptation itself (i.e. resilient result). The latter has also been defined differently as recovery (i.e. bouncing back from a negative change), sustainability (i.e. no change at all) or growth (i.e. improvement) after the exposure to an adverse experience. Two contemporary pain related models merge these concepts into a larger construct featuring stable and modifiable intra- and interpersonal resources, among them self-regulation, optimism, social capital, social skills and coping, as well as the different types of resilient outcomes [13, 14]. These models define resilience as the availability of a set of personal, social or societal protective factors that lead to a favourable outcome despite chronic pain [11, 13, 14]. Resilience is generally associated with less depression and greater mental well-being [12, 15].

Within pain research resilience has been negatively associated with catastrophizing [16] and anxiety [17] and positively with functioning and quality of life [17, 18]. So far, only two studies examined resilience together with disability. Both of them found significant correlations with medium effect sizes. In addition, one of the two studies showed that the association between resilience and disability was mediated by acceptance. However, these studies were only cross-sectional and no conclusions about the prognostic influence of resilience on the course of the disease are possible on the basis of said studies [19, 20].

Coping resources might also act as a protective factor. They are defined as a patient’s potentials in dealing with his/her disease successfully (adaptive coping) [21]. In pain patients adaptive coping is cross-sectionally associated with higher pain-related self-efficacy [22] less depression, anxiety and distress [20, 23, 24], increased functioning and low levels of impairment [25, 26] and less pain [20, 26]. But again, longitudinal studies are scarce. Only a few studies examined the influence of adaptive coping on chronic pain over time. They found that daily coping measures predicted a reduction in pain intensity the following day but they did not include disability [27].

In summary no study to date has examined the impact of protective factors such as resilience and coping resources on pain-related disability in a longitudinal design. Based on earlier cross-sectional studies, we hypothesize that resilience and coping resources can act as protective factors for disability. We therefore included both constructs in a longitudinal predictor analysis alongside the established risk factors depression, somatization and duration of pain. So far there is no evidence for age or gender being risk factors for disability in chronic pain. However, in another analysis we performed on the same sample, gender predicted pain generalization [28]. Also, while age is often measured to describe sample characteristics, it is rarely examined as a predictor of disability in ongoing chronic pain syndromes. In a recent Canadian study age mediated the relationship between pain intensity and disability in a recent cross-sectional Canadian study [29]. We thus included both variables in our analysis to examine any influence they might have on the development of disability.

## Methods

### Study design

We conducted a prospective cohort study on patients reporting CLBP at their primary care practitioner. After inclusion demographic data, pain characteristics and potential risk and protective factors were measured at baseline. Patients were then followed up for 12 months when disability was measured again. The reported data is a pre-specified secondary analysis of a prospective cohort study examining the influence of possible risk- and protective factors on the transition from chronic localized low back pain (CLBP) into chronic widespread pain (CWP) [30].

### Recruitment and data collection

A total of 58 general practitioners (GPs) in the northern part of the state Hesse in Germany consecutively recruited all eligible patients consulting with chronic low back pain over the course of five months. Chronic low back pain was defined as pain in the back area under the costal arch and over the bottom fold on more than half of the days over the past 3 months. Exclusion criteria were low fluency in German (subjectively judged by the GP) dementia, pregnancy and being younger than 18 years. Immediately after the consultation and after written consent was given, the patients were asked to answer a set of questionnaires including a pain drawing. Two independent raters analysed each drawing and categorized it as either CLBP or CWP according to the criteria of the American College of Rheumatology [31]. In case of disagreements, the classification was discussed. Only the patients with CLBP were examined further. Additional questionnaires were mailed to them quarterly until the final assessment after 12 months Patients were offered 10€ per completed questionnaire, giving them the opportunity to earn up to 50€ over the course of the entire study.

### Baseline measurements

#### Pain and sociodemographic characteristics

Pain characteristics such as the duration of pain (5 alternatives: from “onset less than one year ago” up to “onset 5 to 10 years ago”) were assessed with the pain characteristics subscale of the German Pain Questionnaire [32, 33]. In addition to that, patients were asked to mark the painful regions in a pain drawing [34, 35]. For evaluation purposes, the drawing was split into 10 body parts with a stencil (“head”, “back-neck area”, “back-chest area”, “lower back area”, “left/right shoulder and arm”, “left/right leg”, “chest and belly”, “sternum”) [36, 37]. Both the number of pain areas and the combination of certain areas were considered to classify the pain syndrome as either CLBP or CWP according to the ACR-criteria (i.e. pain in the upper and lower part as well as the left and right side of the body = axial skeleton and contralateral quadrants) [31]. In order to evaluate demographic parameters of the baseline sample, the respective subscale of the German Pain Questionnaire was used [32].

#### Pain related disability

At baseline the pain related disability was measured with the Graded Chronic Pain Questionnaire (GCPQ) by von Korff [38]. Patients rated the momentary mean and highest pain intensity during the last three months as well as the pain related disability in work, leisure and daily living on an 11-point Likert-scale. The disability score used for statistical analysis was calculated as the mean disability multiplied by 10 (range 0–100). Patients were classified as low disability with low or high pain intensity (von-Korff-grades 1 and 2) and high disability with moderate or severe limitation (von-Korff-grades 3 & 4). The employed German version of the scale has been shown to be reliable (α = 0.82) and externally valid [39].

#### Depression and somatization

Depression was assessed with the Hospital Anxiety and Depression scale (HADS) [40, 41]. Patients rated their agreement with 7 statements relating to depression on a 4 point Likert-scale. Reliability (α coefficients around .81) and external validity have been thoroughly examined and proven since its first publication [42–44].

Somatization was measured with the German version of the somatization subscale of the Symptom Check-List-90-R (SCL-90-R) [45]. The scale presents 12 somatic symptoms relating to dysfunction in autonomic physical systems. These symptoms often occur in functional disorders but can also be part of real physical diseases. Patients rated how much they suffered from each symptom during the last 7 days on a 5 point-Likert scale. The questionnaire is widely used and its reliability and validity are well documented (e.g. Cronbach’s α for somatization in a primary care sample = 0.83) [46].

#### Resilience and coping resources

Resilience was measured with the Resilience Scale by Wagnild and Young (short version RS-11) [47, 48]. The authors define resilience as the personal resource to stay mentally healthy or rebound quickly despite (chronic) stress and life difficulties. The short economic scale contains 11 items covering the areas “personal competence” as well as “acceptance of self and life”. The items are rated on a 7 point Likert scale and the final score is the mean across all items (range 1–7). Considering Yeung’s contemporary model of resilience, those domains can be linked to some of the stable (mostly ego-resilience) and modifiable (mostly emotional complexity/acceptance) intrapersonal resilience constructs [14]. Therefore the scale can be seen as a more general measurement of resilience. The German version has been shown to be reliable (α = .91) and valid [49] although it has to be pointed out, that the here proclaimed links with Yeung’s model haven’t been explicitly tested yet.

Coping resources were evaluated with the Coping Resources for Back Pain Questionnaire (CRBPQ) [21]. The scale asks for the efficiency of 12 different coping strategies on an 11-point Likert-scale (0 = “not helpful”; 11 = “very helpful”). While the authors intended to form 7 subscales consisting of broader coping areas such as “cognitive strategies”, “knowledge”, “relaxation” and “spirituality”, recent results have suggested the calculation of a mean total score (range 0–10) [50]. The higher the total score the more strategies are rated as helpful. The reliability of the scale has been shown (α = .89) [50].

### Follow-up measurement

At follow up the disability subscale of the German version of the West Haven-Yale Multidimensional Pain Inventory (MPI-D) [51] was used to evaluate disability. The scale consists of 10 items asking for the perceived disability in work, daily activities and social life rated on a 7-point Likert-scale. The calculated score is the mean of those items (range 0–6). The German version has been shown to be reliable (α = .94) and valid [52]. The content of three of the ten MPI-D disability items matches the three items of the GCPQ we used at baseline (disability in everyday activities, disability in work, disability in leisure and social activities). We took those three items, transformed their 7-point Likert-scale into the 11-point format of the GCPQ and then calculated the mean multiplied by 10 as we did for the GCPQ.

### Statistical analysis

Descriptive data of the sample was evaluated using SPSS (Version 21) [53]. All other analyses were carried out with R [54]. We first calculated bivariate spearman correlations for all predictors with each other and with follow-up disability. In addition we calculated partial correlations for each predictor with follow-up disability given baseline disability. We then carried out a multiple regression analysis to evaluate the influence of our hypothesized model. We included baseline disability age, gender, duration of pain, somatization, depression, resilience and coping resources as predictors and disability at follow-up as the dependent variable. Before that, missing data were imputed using the multivariate imputation by chained equations (mice) technique [55]. Imputed values were checked for plausibility by comparing plots of imputed and observed values and plots of their distribution conditional on propensity scores [56]. We then ran our multiple regression model with each of the 20 imputed datasets and pooled the results accordingly [57]. Separate estimates and standard errors of regression coefficients were thus combined to overall estimates with standard errors, confidence intervals, and *p*-values. In addition, we reported pooled goodness-of-fit measures (R^2^) and the Akaike Information Criterion (AIC). Effect sizes of regression coefficients were assessed in terms of Cohen´s f^2^ [58]. The Bonferroni-Holm method for multiple comparisons was used to control the type I error rate [59].

We additionally performed three explorative subgroup analyses to compare subgroups with a) low vs high baseline disability (von-Korff Grades ≤ 2 vs >2) b) duration of pain less vs. more than 2 years, c) patients which did vs. did not transition into widespread pain at follow-up. Tests on interaction were done using the methods described by Altman [60] to check for differences in β-weights between the subgroups.

## Results

### Sample characteristics

**Fig. 1:**
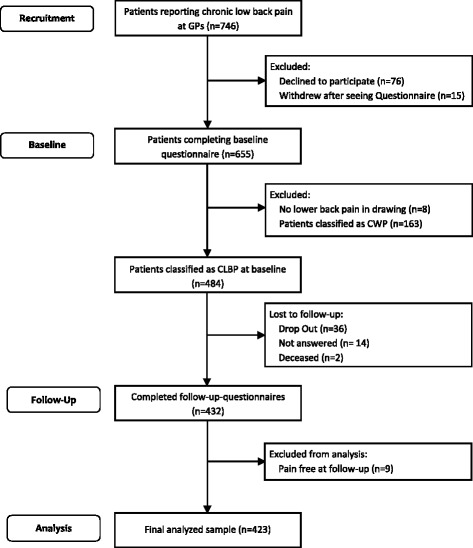
Flowchart of the study sample. *GPs* General Practitioners, *CWP* Chronic Widespread Pain, *CLBP* Chronic Low-Back Pain

A total of 58 GPs participated. During the recruitment period 749 patients with CLBP consulted them with CLBP and 655 gave written consent. Eight patients were later excluded from the study since they reported no low back pain in the drawing and therefore didn’t meet the inclusion criteria. The remaining sample (*n* = 647) was composed of patients with CWP (*n* = 163) and patients with CLBP (*n* = 484). The latter were included in the study and followed up for 12 months. During the follow-up period, 52 patients (11%) were lost: 36 withdrew consent, 2 died and 14 did not send the last questionnaire back (see Fig. 1). We attempted to call each patient not responding to the last questionnaire for six times before we excluded them. The follow-up sample consisted of 432 patients, 89% of the starting sample. Out of those, 320 still reported CLBP in the pain drawing, while 103 had transitioned into CWP and 9 were without any pain. We included all patients with persistent CLBP or those shifting from CLBP into CWP in the analysis (*n* = 423).

Table 1 shows the baseline characteristics of our final sample (*n* = 423). The mean age was 56.6 years (SD = 14.1) and 57.7% of the patients were female. Most of them were German (96.5%) and married (69.3%). About half of them (53.9%) were employed but 34.6% of those were currently on sick leave. The majority (62.4%) of the unemployed participants was on retirement pension and 8.2% were on disability pension. Half of the sample (50.4%) reported that the onset of pain was more than 10 years ago and more than half of the sample (53.7%) suffered from a high degree of disability (von-Korff grades 3 and 4) at baseline. Clinically relevant depressive symptoms (HADS > 11) were reported by 22.2% of the patients.

**Table 1:** Sample characteristics

Characteristics	Final sample (*n* = 423)	Missing n (%)
Female gender, n (%)	244 (57.7)	0
Age in years, mean (SD)	56.6 (14.1)	0
German nationality, n (%)	408 (96.5)	12 (2.8)
Marital status, n (%)		1 (0.2)
Single	24 (5.7)	
unmarried with partner	23 (5.4)	
married	293 (69.3)	
divorced or separated	47 (11.1)	
widowed	35 (8.3)	
Education graduation in years, n (%)		2 (0.5)
9 years	216 (51.1)	
10 years	130 (30.7)	
12–13 years	64 (15.1)	
Other	10 (2.4)	
No graduation	1 (0.2)	
Currently working (employed), n (%)	228 (53.9)	1 (0.2)
Amount of them currently on sick leave, n (%)	79 (34.6)	4 (1.8)
Days on sick leave in the past 3 months, mean (SD)	10.98 (21.58)	21 (9.2)
Reason for not working, n (%)		2 (1.0)
unemployed	16 (8.2)	
pupil/student	1 (0.5)	
housewife	30 (15.5)	
disability pension	16 (8.2)	
retirement pension	121 (62.4)	
other type of pension	8 (4.1)	
Number of pain areas at baseline, mean (SD)	3.08 (1.60)	0 (0)
Time since onset of back pain, n (%)		0 (0)
< 1 year	58 (13.7)	
1 to 2 years	37 (8.7)	
2 to 5 years	57 (13.5)	
5 to 10 years	58 (13.7)	
> 10 years	213 (50.4)	
Chronic pain grade (Von-Korff), n (%)		32 (7.6)
Grade 1	62 (14.7)	
Grade 2	102 (24.1)	
Grade 3	112 (26.5)	
Grade 4	115 (27.2)	
Korff-disability score (Range 0–100), mean (SD)	49.4 (23.8)	8 (1.9)
Depression (HADS, Range 0–21), mean (SD)	8.00 (3.11)	6 (1.4)
Clinically relevant depression, (HADS > 10), n (%)	94 (22.2)	
Somatization (SCL-90-R, Range 0–4), mean (SD)	0.83 (0.48)	14 (3.3)
Resilience (RS11, Range 1–7), mean (SD)	5.30 (1.16)	25 (5.9)
Coping resources (CRBPQ, Range 0–10), mean (SD)	5.86 (2.12)	17 (4.0)

*SD* Standard Deviation, *HADS* Hospital Anxiety and Depression Scale, *SCL-90-R* Symptom Checklist-90-R, *RS11* Resilience Scale, *CRBPQ* Coping Resources for Back Pain Questionnaire

### Associations between predictors at baseline and with disability at follow-up

The bivariate associations between all predictors at baseline are shown in Table 2. Disability was positively associated with somatization and depression and negatively with resilience. Somatization and depression were positively associated with each other and negatively with resilience and coping resources. In addition only somatization also correlated with the pain duration. Resilience and coping also correlated positively with each other. The pain duration correlated negatively with coping resources and positively with age. Gender was not associated with any of the other predictors.

**Table 2:** Pairwise spearman correlations (ρ) of baseline variables (predictors)

	Baseline disability	Age	Somatization	Depression	Duration	Coping resources	Resilience
Baseline disability	1						
Age	0.03	1					
Somatization	0.43^‡^	−0.02	1				
Depression	0.31^‡^	0.09	0.36^‡^	1			
Duration of pain	0.01	0.15^†^	0.11^*^	0.07	1		
Coping resources	– 0.08	– 0.01	−0.13^*^	– 0.31^‡^	– 0.14^†^	1	
Resilience	– 0.12*	– 0.04	−0.21^‡^	– 0.48^‡^	– 0.05	0.40^‡^	1
Gender	−0.08	0.09	0.08	0.04	– 0.06	0.08	<0.01

*p < .05; ^**†**^ *p* < .01; ^‡^ *p* < .001

Bivariate spearman correlations and partial correlations (given baseline disability) between the predictors and follow up disability are shown in Table 3. Besides gender all predictors were associated with follow-up disability. Baseline disability somatization and depression had the highest associations (medium effect sizes) [61]. The correlations between pain duration and disability as well as age and disability were also significant but with smaller effect sizes. Resilience and coping resources negatively correlated with follow-up disability however the associations were small.

**Table 3:** Spearman correlations and partial correlations (controlled for baseline disability) of baseline predictors with disability at follow-up

	ρ	ρ-partial
Baseline disability	0.46^‡^	
Age	0.26^‡^	0.28^‡^
Somatization	0.40^‡^	0.23^‡^
Depression	0.35^‡^	0.25^‡^
Time since onset of pain	0.14^†^	0.13^†^
Coping resources	−0.15^†^	−0.12^*^
Resilience	−0.21^‡^	−0.18^‡^
Gender	0.02	0.08

*p < .05; ^**†**^ *p* < .01; ^‡^ *p* < .001

Table 4 shows the associations between all predictors and disability after 1 year (multiple regression). The total degree of explained variance was 35% (*R* ^*2*^ = 0.35). After controlling for alpha inflation with the Bonferroni-Holm method baseline disability (β = 0.34, *f* ^*2*^ = 0.126), age (β = 0.41, *f* ^*2*^ = 0.075) and somatization (β = 9.71, *f* ^*2*^ = 0.039) were significantly associated with disability at follow-up. Judging from the effect sizes, somatization had the highest impact out of all the psychological constructs but in general the effect size of all predictors besides baseline disability was small [61].

**Table 4:** Associations of predictors with follow-up disability, using multiple regression analysis

Explanatory Variables	β	95% CI	β_Stand_	*p* – value (β)	Cohen’s *f* ^*2*^
Baseline disability	0.34	0.25, 0.44	0.33	< .001*	0.126
Age	0.41	0.26, 0.55	0.23	< .001*	0.075
Somatization	9.71	4.76, 14.67	0.19	< .001*	0.039
Depression	0.83	0.01, 1.64	0.10	.046	0.011
Time since onset of pain	1.19	−0.21, 2.59	0.07	.096	0.007
Coping resources	– 0.58	– 1.64, 1.64	– 0.05	.280	0.003
Resilience	– 0.83	– 2.96, 1.31	– 0.04	.446	0.002
Gender	0.64	– 3.47, 4.76	0.01	.759	<0.001
AIC	3766.12				
*R* ^*2*^	0.35				

β = regression coefficient; βstand = standardized regression coefficient; *CI* confidence interval

Cohen’s *f* ^*2*^: 0.02 = small effect size, 0.15 = medium effect size, 0.35 = large effect size

*p < Bonferroni-Holm adjusted *α* (1^st^ test: *α* = 0.05/8 = 0.006, 2^nd^ test: *α* = 0.05/7 = 0.007; 3^rd^ test: α = 0.05/6 = 0.008…)

In the subgroup comparison of patients with low vs. high baseline disability disability, age and somatization predicted follow-up disability in both groups equally (see Additional file 1 for all subgroup comparisons). In the comparison of patients with different pain durations, somatization descriptively had a higher association with follow-up disability in patients suffering for less than 2 years (Cohen’s ƒ = 0.117, *p* = .004) than in patients suffering for more than 2 years (Cohen’s ƒ = 0.025, *p* = .007). However, while noticeable, the statistical test for that effect was not significant (interaction-*p* = .07). Age and somatization also predicted disability in patients without transition into CWP but not in patients with transition into CWP. This descriptive finding however was also not confirmed by the tests for significance (*p*-values for interaction = .296 and .117). However, since all the subgroup analyses were done exploratory they might not have been adequately powered. Finally, neither resilience nor coping resources predicted disability in any of the subgroups.

## Discussion

To our knowledge this is the first study to examine the influence of resilience and coping resources on disability in a longitudinal design. It examined a sample that consisted entirely of primary care patients with CLBP and followed them over the course of one year.

Each of the predictors except gender bivariately correlated with follow-up disability. However when all predictors were examined simultaneously in a multiple regression analysis to address intercorrellation only baseline disability, age and somatization predicted disability. Higher values in those three predictors led to stronger disability at follow up rendering them risk factors. Resilience and coping resources did not predict disability in contrast to our assumption.

Although disability at baseline was by far the strongest predictor for disability after one year the effect size was only in the medium range. This shows that individual changes in disability are still possible and that there is a chance for prediction even in patients with very chronic conditions.

In contrast to our assumption resilience did not predict follow-up disability. This contradicts earlier cross-sectional findings where resilience was associated with disability [19 20]. We found a small cross sectional association between resilience and disability in our data as well (*r* = −.12), but this correlation did not carry over to the longitudinal multivariate analysis. There are differences in the study samples of those studies and our study that might explain the contrasting results. Our study included many patients that had been suffering from chronic pain already for a longer time (over 60% for more than 5 years) and were highly disabled at baseline already (over 50% had a von-Korff grade of 3 and 4), reflecting a high initial degree of chronification. This is in contrast to the study by Ramirez-Maestre et al. [20] where more than 60% of the sample were suffering from chronic pain for less than 2 years. Additionally, their measurement of disability differed from ours making it difficult to compare the initial levels of disability. The report by Ruiz-Párraga [19] lacks data which is essential for a comparison (mean disability, pain duration). Both studies however used the same measurement of resilience as we did. In comparison to the study by Ramirez-Maestre, the high proportion of disabled patients suffering for a longer time in our sample could thus be responsible for the lack of association between resilience and disability. Even when we performed additional subgroup comparisons, resilience did not predict disability in patients with less disability (von-Korff ≤ 2) or shorter pain durations (≤2 years). Therefore, any effect that resilience could have on disability might be limited to acute or subacute stages of the chronification process. The same applies to coping resources which we also expected to act as a protective factor. Although there are cross sectional associations between coping resources and disability in the literature we could not confirm them in the longitudinal multivariate analysis. We therefore conclude that resilience and coping resources might not have the potential to act as predictors for favourable courses of disability – at least not in patients suffering from chronified low back pain.

In our study somatization was the only psychosocial construct which predicted disability. This adds to existing evidence of somatization being a risk factor for disability and chronification [5, 7]. But how exactly could somatization contribute to higher levels of disability? The scale we used to measure somatization presents 12 somatic symptoms (e.g. head-, chest- and muscular pain in different areas, dizziness, heavy breathing, feelings of weakness or heaviness, nausea) relating to dysfunction in autonomic physical systems and asks patients how much they have been suffering from these symptoms. Therefore a higher score reflects suffering from more symptoms and to a higher degree. There are several possible explanations how this can relate to stronger disability. First, people who suffer from more somatic symptoms are known to feel increased fatigue and rest more which will unintentionally contribute to further decline in functional ability and feelings of disability [62–64]. Second, people who experience more symptoms might feel more helpless and pessimistic which is associated with depression, passive coping (i.e. rest) and functional impairment [26, 65]. Finally, somatization could be a facet of Fibromyalgia which also includes pain spreading and fatigue. While we can neither confirm nor deny the first two possible explanations, or data contradicts the third. In one of the additional subgroup analysis, we compared patients transitioning into CWP with those who did not. While somatization was significantly associated with follow-up disability in the subgroup which did not transition into CWP there was no association in the subgroup which did (see Additional file 1). However, the test for interaction between the two groups for the regression of somatization on disability was not significant although descriptively the differences where apparent.

There is robust evidence that depression is a risk factor for disability and chronification [66, 67]. The fact that it did not predict disability in our analysis contrasts these established findings. The most suitable explanations for this are methodological. First, we used the Bonferroni-Holm method to address alpha inflation in the multiple regression. Although addressing alpha inflation is recommended when interpreting multiple comparisons [68] most of the studies actually do not apply any form of *α*-correction. The *p*-value for depression was .046. Without *α*-correction, we would have concluded that depression was in fact a predictor for disability. Second, as we also discussed for resilience, most studies on risk factors examined (sub-)acute pain [66] and the influence of depression on disability might be reduced in more chronified samples. Third, the majority of studies that examined risk factors for disability and chronification included depression but not somatization [66, 67]. This is important since the results of a multiple regression always depend on the selection of predictors [69] and there is intercorrellation between both constructs [50]. Therefore the inclusion of somatization besides depression can change the results. Two extensive and established reviews of predictors for disability [66, 67], found only one study that also included somatization [70]. The reported data indicates that somatization can have an equal influence on disability as depression. Without somatization in our analysis, we expect that depression would have had a bigger and maybe also significant impact in line with other studies. But this would have been due to confounding. Based on our findings, we now suggest, that future studies should examine somatization together with depression to re-evaluate the influence of depression on disability.

In population based samples the prevalence for CLBP is higher in older patients [1, 71–73] and younger patients seem to recover slightly better under multidisciplinary therapy [74]. In samples composed only of chronic low back pain patients however, there is usually no cross-sectional relationship between age and disability [75]. In our study, higher age also did not correlate with disability at baseline but it predicted higher disability after one year. There are several possible explanations for this finding. First, older patients might have less favourable courses of pain. For example they might develop pain in more regions and therefore also experience more disability. However, when we examined the transition into CWP in another analysis of the same sample, pain generalization was not predicted by age [28]. We can therefore rule out that the age-disability relationship is confounded by pain generalization. Second, multimorbidity increases with age which leads to additional disability [76, 77]. Even though patients were asked to rate the disability caused by the pain, it might not have been possible for them to distinguish disability caused by pain or by other comorbidities, especially when their condition has been chronic for years. We can neither confirm nor deny this assumption from our data, since we did not measure the development of comorbidities over time.

### Strengths and limitations

This study has some limitations. First due to the fact that it is a secondary analysis of data collected for a slightly different purpose (i.e. examining the impact of risk- and protective factors on pain generalization), some constructs have not been measured in the most suitable way. The scale we used to measure coping resources asks for their efficiency but it still lacks evidence that this also reflects their actual application [21]. So even if patients rate coping resources as helpful, it does not imply that they use them often. Second, disability at baseline and at follow-up was measured with different questionnaires. This impedes the detection of change in disability over time which is a very unfortunate and limiting circumstance. This was caused by the fact that the project was part of a larger multiproject consortium where a mandatory core set of questionnaires for each point in time was agreed upon at funding. In addition since we collect our data in the field and rely on the voluntary participation of GPs and their patients, we have to carefully avoid putting too much workload on them. Therefore we opted against the inclusion of yet another scale. We alleviated this limitation as best as we could by transforming the follow-up questionnaire to fit the baseline measure (see methods section). But it is still not the same as using the same scale. Third, to achieve adequate statistical power of the multiple regression we had to limit the amount of predictors. This led to the exclusion of some constructs for which there is evidence or at least indication they can act as predictors such as catastrophizing, fear-avoidance, self-efficacy and acceptance [27]. This is especially important since the results of a multiple regression can depend on the selection of variables. Future studies should consider the proposed additional constructs to allow for a more complete model of prediction. Finally, our sample consisted to a large degree (>50%) of patients with high disability. This differs from findings in German population based studies where only 10% are highly disabled [1]. Thus, individuals with higher resilience and less chronified pain might not have been included. This makes our results less comparable to the general population and restricts the external validity of our findings.

Despite these limitations this is one of the biggest (*n* = 423) longitudinal studies about chronic low back pain in primary care to date and the only study to include possible protective factors such as resilience and coping resources. Another strength is the high participation rate at follow up (89%). This was achieved by putting a lot of effort in caretaking of the sample (e.g. Christmas cards, appreciative letters).

## Conclusions

The results of this study suggest that neither resilience nor coping resources can act as predictors for favourable courses of disability in CLBP patients. Therefore interventions aimed at improving resilience and coping resources might not be suitable. However, due to the sample composition, this conclusion is limited to primary care patients suffering from much chronified pain syndromes. We cannot rule out, that a) resilience and coping resources can influence the development of disability in patients suffering from acute or subacute low back pain and b) more specific constructs such as pain-acceptance and self-efficacy might be more beneficial in predicting disability. Future studies should take this into account and adjust their inclusion criteria and/or predictor selection accordingly.

## Additional file

Additional file 1: Table S1. Subgroup comparison: Baseline von-Korff disability ≤ 2 vs. > 2. **Table S2.** Subgroup comparison: Duration of pain ≤ 2 years vs. > 2 years. **Table S3.** Subgroup comparison: Transition into widespread pain at follow-up No vs. Yes. (DOCX 29 kb)

## Abbreviations

AIC: Akaike information criterion
BL: Baseline
CI: Confidence interval
CLBP: Chronic low back pain
CRBPQ: Coping resources for back pain questionnaire
CWP: Chronic widespread pain
GP: General practitioner
GSE: General self-efficacy
HADS: Hospital anxiety and depression scale
MPI-D: Multiphasic pain inventory German version
RS-11: Resilience scale short version
SD: Standard deviation
