# Supplementary material for: Psychological risk and protective factors for disability in chronic low back pain – a longitudinal analysis in primary care
Source: BMC Musculoskelet Disord. 2017 Mar 20;18:114. doi: 10.1186/s12891-017-1482-8 (PMC5360090; doi:10.1186/s12891-017-1482-8)
Supplement: Additional file 1: Table S1. — Subgroup comparison: Baseline von-Korff disability ≤ 2 vs. > 2. Table S2. Subgroup comparison: Duration of pain ≤ 2 years vs. > 2 years. Table S3. Subgroup comparison: Transition into widespread pain at follow-up No vs. Yes. (DOCX 29 kb) [file 12891_2017_1482_MOESM1_ESM.docx]

**Supplemental material**

**Table 1 Subgroup comparison: Baseline von-Korff disability ≤ 2 vs. > 2**

|  | Split Korff-Disability Grade ≤ 2 (n = 176) | | | | | Split Korff-Disability Grade > 2 (n = 247) | | | | |  |
| --- | --- | --- | --- | --- | --- | --- | --- | --- | --- | --- | --- |
|  | **β** | **β_Stand_** | **95% CI** | ***p* – value** | **Cohen’s *f^2^*** | **β** | **β_Stand_** | **95% CI** | ***p* – value** | **Cohen’s *f^2^*** | **Interaction *p*-value*** |
| BL disability | 0.32 | 0.23 | 0.12; 0.52 | .002 | 0.067 | 0.17 | 0.13 | 0.01; 0.33 | .040 | 0.018 | .252 |
| Age | 0.45 | 0.28 | 0.23; 0.68 | <.001 | 0.102 | 0.35 | 0.21 | 0.15; 0.55 | <.001 | 0.054 | .498 |
| Somatization | 13.3 | 0.21 | 4.19; 22.41 | .004 | 0.053 | 8.33 | 0.19 | 2.43; 14.22 | .006 | 0.035 | .365 |
| Depression | 0.71 | 0.09 | -0.59; 2.01 | .283 | 0.009 | 0.96 | 0.13 | -0.09; 2.0 | .072 | 0.015 | .767 |
| Duration of pain | 0.74 | 0.05 | -1.4; 2.88 | .496 | 0.003 | 1.48 | 0.1 | -0.39; 3.36 | .120 | 0.010 | .606 |
| Coping resources | 0.08 | 0.01 | -1.61; 1.76 | .928 | <0.001 | -0.9 | -0.09 | -2.31; 0.51 | .210 | 0.007 | .380 |
| Resilience | -2.86 | -0.14 | -6.27; 0.56 | .100 | 0.022 | 0.62 | 0.03 | -2.14; 3.38 | .658 | 0.001 | .117 |
| Gender | -2.00 | -0.04 | -8.55; 4.54 | .546 | 0.003 | 2.11 | 0.05 | -3.28; 7.5 | .441 | 0.003 | .338 |
| AIC | 1565.80 |  |  |  |  | 2202.95 |  |  |  |  |  |
| *R^2^* | 0.27 |  |  |  |  | 0.20 |  |  |  |  |  |

BL = Baseline; β = regression coefficient; CI = confidence interval; Cohen’s *f^2^*: 0.02 = small effect size, 0.15 = medium effect size, 0.35 = large effect size

* Test for interaction between group and the regression of each variable on follow-up disability. No interaction means that the respective predictor is not differently associated with disability in the two groups.

**Table 2 Subgroup comparison: Duration of pain ≤ 2 years vs. > 2 years**

|  | **Duration of pain ≤ 2 years (*n* = 95)** | | | | | **Duration of pain > 2 years (n = 328)** | | | | |  |
| --- | --- | --- | --- | --- | --- | --- | --- | --- | --- | --- | --- |
|  | **β** | **β_Stand_** | **95% CI** | ***p* – value** | **Cohen’s *f^2^*** | **β** | **β_Stand_** | **95% CI** | ***p* – value** | **Cohen’s *f^2^*** | **Interaction *p*-value*** |
| BL disability | 0.29 | 0.28 | 0.07; 0.5 | .009 | 0.086 | 0.37 | 0.35 | 0.26; 0.48 | <.001 | 0.143 | .505 |
| Age | 0.28 | 0.18 | -0.02; 0.58 | .066 | 0.042 | 0.47 | 0.26 | 0.3; 0.64 | <.001 | 0.098 | .270 |
| Somatization | 20.64 | 0.34 | 7.0; 34.27 | .004 | 0.117 | 7.4 | 0.15 | 2.01; 12.79 | .007 | 0.025 | .073 |
| Depression | 0.29 | 0.03 | -1.76; 2.34 | .780 | 0.002 | 0.91 | 0.12 | 0.02; 1.8 | .046 | 0.014 | .582 |
| Duration of pain | 4.28 | 0.08 | -5.29; 13.84 | .377 | 0.01 | 0.61 | 0.02 | -2.36; 3.57 | .687 | 0.001 | .467 |
| Coping resources | 0.04 | <0.01 | -2.19; 3.42 | .973 | 0.001 | -0.76 | -0.06 | -1.99; 0.48 | .230 | 0.005 | .536 |
| Resilience | -2.16 | -0.09 | -7.73; 3.42 | .442 | 0.012 | -0.42 | -0.02 | -2.74; 1.89 | .719 | 0.001 | .566 |
| Gender | 5.5 | 0.11 | -3.83; 14.83 | .244 | 0.017 | -0.31 | -0.01 | -4.9; 4.29 | .896 | <0.001 | .268 |
| AIC | 858.51 |  |  |  |  | 2916.71 |  |  |  |  |  |
| *R^2^* | 0.35 |  |  |  |  | 0.35 |  |  |  |  |  |

BL = Baseline; β = regression coefficient; CI = confidence interval; Cohen’s *f^2^*: 0.02 = small effect size, 0.15 = medium effect size, 0.35 = large effect size

* Test for interaction between group and the effect of a variable on disability. No interaction means that the respective predictor is not differently associated with disability in the two groups.

**Table 3 Subgroup comparison: Transition into widespread pain at follow-up No vs. Yes**

|  | **No transition into widespread pain (n = 320)** | | | | | **Transition into widespread pain (n = 103)** | | | | |  |
| --- | --- | --- | --- | --- | --- | --- | --- | --- | --- | --- | --- |
|  | **β** | **β_Stand_** | **95% CI** | ***p* – value** | **Cohen’s *f^2^*** | **β** | **β_Stand_** | **95% CI** | ***p* – value** | **Cohen’s *f^2^*** | **Interaction *p*-value*** |
| BL disability | 0.30 | 0.29 | 0.19; 0.41 | <.001 | 0.095 | 0.49 | 0.49 | 0.28; 0.7 | <.001 | 0.276 | .114 |
| Age | 0.43 | 0.24 | 0.26; 0.6 | <.001 | 0.081 | 0.25 | 0.15 | -0.05; 0.55 | .101 | 0.034 | .296 |
| Somatization | 11.22 | 0.2 | 4.87; 17.57 | <.001 | 0.044 | 2.99 | 0.07 | -5.25;11.23 | .472 | 0.007 | .117 |
| Depression | 0.98 | 0.12 | 0.02; 1.95 | .046 | 0.015 | 0.23 | 0.03 | -1.33; 1.79 | .773 | 0.002 | .412 |
| Duration of pain | 1.03 | 0.06 | -0.58; 2.64 | .209 | 0.005 | 0.62 | 0.03 | -2.48; 3.73 | .691 | 0.003 | .812 |
| Coping resources | -0.15 | -0.01 | -1.4; 1.11 | .82 | <0.001 | -1.48 | -0.14 | -3.48; 0.52 | .145 | 0.029 | .263 |
| Resilience | -0.66 | -0.03 | -3.25; 1.93 | .615 | 0.001 | -1.4 | -0.07 | -5.1; 2.3 | .454 | 0.008 | .746 |
| Gender | 0.88 | 0.02 | -3.93; 5.69 | .719 | <0.001 | -1.39 | -0.03 | -9.78; 6.99 | .742 | 0.002 | .641 |
| AIC | 2870.21 |  |  |  |  | 897.55 |  |  |  |  |  |
| *R^2^* | 0.31 |  |  |  |  | 0.43 |  |  |  |  |  |

BL = Baseline; β = regression coefficient; CI = confidence interval; Cohen’s *f^2^*: 0.02 = small effect size, 0.15 = medium effect size, 0.35 = large effect size

* Test for interaction between group and the effect of a variable on disability. No interaction means that the respective predictor is not differently associated with disability in the two groups.
